# In vitro activities of Eravacycline against 336 isolates collected from 2012 to 2016 from 11 teaching hospitals in China

**DOI:** 10.1186/s12879-019-4093-1

**Published:** 2019-06-10

**Authors:** Chunjiang Zhao, Xiaojuan Wang, Yawei Zhang, Ruobing Wang, Qi Wang, Henan Li, Hui Wang

**Affiliations:** Department of Clinical Laboratory, Peking University People’s Hospital, Beijing, 100044 China

**Keywords:** Eravacycline, Tigecycline, Carbapenem resistant *Enterobacteriaceae* bacteria, *Acinetobacter baumannii*, Antibiotic resistance

## Abstract

**Background:**

In China multidrug-resistant bacteria pose a considerable threat to public health. Antimicrobial resistance has weakened the effectiveness of many medicines widely used today. Thus, discovering new antibacterial drugs is paramount in the effort to treat emerging drug-resistant bacteria.

**Methods:**

Eravacycline, tigecycline and other clinical routine antibiotics were tested by reference broth micro-dilution method against 336 different strains collected from 11 teaching hospitals in China between 2012 and 2016. These isolates included *Enterobacteriaceae*, non-fermentative, *Staphylococcus* spp., *Enterococcus,* and a number of fastidious organisms. The strains involved in this study possess the most important drug resistance characteristics currently known in China. Drug resistant bacteria such as those producing extended spectrum β-lactamases (ESBL) and carbapenemases (KPC-2 and NDM-1), and those exhibiting colistin resistance (*mcr*-1) and tigecycline were included in this study. Additionally, methicillin-resistant *Staphylococcus aureus* (MRSA), vancomycin-resistant *enterococci* (VRE), β-lactamase positive *Haemophilus influenzae*, and penicillin resistant S*treptococcus pneumoniae* (PRSP) were also included.

**Results:**

Eravacycline exhibited good efficacy against all the strains tested, especially for organisms with ESBLs, carbapenemases, and *mcr*-1 gene compared with tigecycline and other antibiotics tested. The MIC values of eravacycline against carbapenemase producing *Enterobacteriaceae* and OXA-23-producing *A. baumannii* were much lower than the MIC values of other antibiotics. MRSA, VRE, β-lactamase positive *Haemophilus influenza*, and PRSP were sensitive to eravacycline in every strain tested. Furthermore, in most strains tested, the MICs of eravacycline were two to four-fold lower than the MICs of tigecycline.

**Conclusions:**

Eravacycline has shown potent antibacterial activity against common and clinically important antibiotic-resistant pathogens. The MIC distribution of eravacycline was generally lower than that of tigecycline which demonstrates that this new drug is potentially more effective than the existing medications.

**Electronic supplementary material:**

The online version of this article (10.1186/s12879-019-4093-1) contains supplementary material, which is available to authorized users.

## Background

In China, microbial resistance to presently administered antimicrobial agents is increasing steadily owing to the emergence of novel resistance mechanisms in the microbes [1, 2]. Multidrug-resistant bacterium causes a considerable threat to public health. Antimicrobial resistance weakened the effectiveness of many medicines widely used today [3]. Thus discovering new antibacterial drugs are required to combat the threat of these emerging resistant bacteria. Eravacycline (TP-434 or 7-fluoro-9-pyrrolidinoacetamido-6-demethyl-6-deoxytetracycline) is a novel broad-spectrum synthetic tetracycline antibiotic being developed for the treatment of severe life-threatening infections, including those that are resistant to current broad-spectrum antibiotics [4]. Eravacycline has already been proven effective against some clinically important antibiotic-resistant pathogens, including gram-positive and gram-negative aerobic and anaerobic pathogens [5, 6]. Moreover, eravacycline was found to be safer and more effective than carbapenems in patients with complicated intra-abdominal infection (cIAI) during global phase 3 clinical trials (NCT01844856 and NCT02784704) [5, 7]. Additionally, there is a clinical development plan in place to introduce it into China to address bacterial drug resistance. The targets of eravacycline include complicated intra-abdominal infection (cIAI), complicated urinary tract infection (cUTI), and pulmonary infections caused by other susceptible pathogens. Tigecycline is a relatively new competing drug for eravacycline, imipenem, meropenem, and colistin in the treatment of carbapenem-resistant *Enterobacteriaceae.* The present study was designed to evaluate the in vitro activities of eravacycline against panels of clinical bacterial pathogens, with or without remarkable resistance factors, which were collected in recent years and were similar to pathogenic bacteria that this drug was designed to treat. This study was designed to prove the in-vitro efficacy of eravacycline (presented by minimum inhibitory concentration, MIC) against major target pathogens in China, which will be used to support further clinical development of eravacycline within China.

## Methods

In the present study, a total of 336 different clinical isolates, were routinely collected from 11 teaching hospitals representing the south, north, northwest, east, and middle regions of mainland China between 2012 and 2016, and tested (list of the hospitals can be found in Additional file 1). After re-identification with the typical biochemical reaction of each organism, the strains were stored in a Microbank tube and placed in a refrigerator at − 80 degrees Celsius before test. All organisms and their associated drug resistance factors are detailed in Table 1. MIC measurements were performed via the reference broth microdilution method as described by the Clinical and Laboratory Standards Institute (CLSI) M7-A9 (2012) [8]. *Escherichia coli* ATCC 25922 and *Pseudomonas aeruginosa* ATCC 27853 were utilized as quality controls in MIC testing of gram-negative bacteria. *Staphylococcus aureus* ATCC 29213 and *Enterococcus faecalis* ATCC 29212 were utilized as quality controls in MIC testing of gram-positive bacteria. *Streptococcus pneumoniae* ATCC 49619, *Haemophilus influenzae* ATCC 49247 and *Haemophilus influenzae* ATCC 49766 were used as quality controls during MIC testing of the fastidious organisms. Tigecycline, the major comparator for eravacycline, imipenem, meropenem and colistin to treat carbapenem-resistant *Enterobacteriaceae* and *Acinetobacter baumannii*, were selected in the panel of antibiotics to be tested. We evaluated eravacycline with a gradient concentration of 0.002–16 mg/L against common clinical gram-negative bacilli, gram-positive cocci, and fastidious organisms collected from our previous studies [9–13], including *Enterobacteriaceae* (*Klebsiella pneumoniae, Escherichia coli, Enterobacter cloacae*), *Acinetobacter baumannii*, *Stenotrophomonas maltophilia*, *Staphylococcus aureus*, *Staphylococcus epidermidis*, *Staphylococcus haemolyticus*, *Staphylococcus hominis*, *Enterococcus faecalis*, *Enterococcus faecium*, *Streptococcus pneumoniae* and *Haemophilus influenzae*. Antibiotic solutions for susceptibility testing were freshly prepared according to the manual of CLSI [8]. A scatter plot of eravacycline versus tigecycline was drawn for each species of bacteria, to reveal the relationship between the two antibiotics in different organisms. All the results related to resistant genes were readily available, directly from our previous researches [12–14]. Statistical analyses and data visualization were done with R (version 3.4.4) and ggplot2 package (version 2.2.1).

**Table 1:** The strains involved in this study and antibiotic resistance characteristics of the strains

Group	Identification	Resistance features	Number
Enterobacteriaceae	*Klebsiella pneumoniae*	ESBL	10
		Tigecycline resistant	13
		*kpc*-2 positive	9
		NDM-1 positive	3
		*mcr*-1 positive	4
		Sensitive ^a^	10
	*Escherichia coli*	ESBL	10
		*mcr*-1, NDM-5	5
		Carbapenem resistant	10
		Sensitive ^a^	10
	*Enterobacter cloacae*	ESBL	6
		Carbapenem resistant	1
		Sensitive ^a^	22
Non-fermentive	*Acinetobacter baumanii*	OXA-23 positive	21
		Tigecycline resistant	9
		Sensitive ^a^	9
	*Stenotrophomonas maltophilia*	Sensitive ^a^	29
Staphylococcus sp.	*Staphylococcus aureus*	MRSA	15
		MSSA	6
	*Staphylococcus epidermidis*	MRCoNS	10
		MSCoNS	10
	*Staphylococcus haemolyticus*	MRCoNS	8
		MSCoNS	1
	*Staphylococcus hominis*	MRCoNS	6
		MSCoNS	4
Enterococcus	*Enterococcus faecalis*	Sensitive ^a^	10
	*Enterococcus faecium*	VRE	3
		Sensitive ^a^	8
Fastidious	*Haemophilus influenzae*	β-lactamase negative	10
		β-lactamase positive	10
	*Streptococcus pneumoniae*	PRSP	10
		PSSP	10

^a^: Sensitive strains referred to strains do not have specific resistance characteristics such as ESBL, carbapenem resistance, polymyxin resistance and glycopeptide resistance

## Results

In vitro activity of eravacycline was evaluated against 336 strains of clinically significant species, with many exhibiting resistance factors (Table 1). In most of the strains tested, the MIC_50_ and MIC_90_ values for eravacycline were lower than that of tigecycline and other comparable antibiotics tested for each organism/phenotypic group. Furthermore, eravacycline was highly effective against all of the organisms tested, regardless of resistance factors.

For *Enterobacteriaceae* bacteria, the MIC values of eravacycline varied with the resistance characteristics, especially for *K. pneumoniae*. The MIC_50_ values of eravacycline against *E. cloacae* and *E. coli* were much lower than the values of other comparable drugs, especially in strains with resistance phenotypes (Table 2). For *K. pneumoniae*, the MIC distribution of eravacycline differed depending on the drug resistance features. *K. pneumoniae* strains which were ESBL-positive (*n* = 10), *kpc*-2-positive (*n* = 9) and NDM-1-positive (*n* = 3), had similar MIC distributions. The MIC_50_ value of eravacycline against strains with the above three resistance mechanisms is 0.5 mg/L, and the MIC90 values were 1 mg/L, 2 mg/L and 1 mg/L respectively.

**Table 2:** MIC distribution of Eravacycline and relevant antibiotics against *E. coli* and *E. cloacae* of different resistance characteristics

Organism	Antibiotics	Carbapenem resistant ^a^			ESBL			Sensitive ^b^		
		MIC_50_	MIC_90_	Range	MIC_50_	MIC_90_	Range	MIC_50_	MIC_90_	Range
*E.coli*	Eravacycline	0.5	1	0.064–2	0.125	0.25	0.064–0.25	0.064	0.125	0.064–0.25
	Tigecycline	1	2	0.25–4	0.25	0.5	0.25–0.5	0.25	0.25	0.125–0.5
	Piperacillin/Tazobactam	256	256	2–256	2	8	1–256	1	2	0.5–2
	Cefoxitin	256	256	64–256	8	32	4–32	2	4	2–8
	Ceftazidime	256	256	0.5–256	32	64	16–128	0.064	0.25	0.064–0.25
	Cefoperazone/Sulbactam	256	256	8–256	16	32	8–256	0.25	1	0.064–4
	Ceftriaxone	256	256	2–256	256	256	64–256	0.032	0.064	0.016–0.064
	Cefotaxime	256	256	4–256	256	256	64–256	0.032	0.064	0.032–0.064
	Cefepime	64	256	0.25–256	32	64	8–128	0.016	0.032	0.016–0.064
	Ertapenem	32	32	16–32	0.125	0.25	0.016–1	0.016	0.016	0.016–0.016
	Imipenem	8	32	8–64	0.125	0.125	0.125–1	0.125	0.125	0.064–0.125
	Meropenem	8	32	4–32	0.032	0.064	0.016–0.064	0.016	0.016	0.016–0.016
	Amikacin	4	256	0.5–256	2	4	1–8	2	2	1–4
	Minocycline	8	16	0.5–16	1	8	0.5–16	1	2	0.5–8
	Ciprofloxacin	64	64	0.064–64	32	64	0.25–64	4	32	0.016–32
	Levofloxacin	16	64	0.125–128	16	32	0.5–64	8	8	0.032–16
	Moxifloxacin	16	32	0.5–64	16	32	0.5–64	8	16	0.032–16
*E.cloacae*	Eravacycline	0.5	0.5	0.5–0.5	0.25	0.5	0.125–0.5	0.5	0.5	0.125–1
	Tigecycline	2	2	2–2	1	1	0.125–2	0.5	2	0.5–2
	Piperacillin/Tazobactam	256	256	256–256	4	4	2–8	2	64	0.5–256
	Cefoxitin	256	256	256–256	8	32	4–256	256	256	64–256
	Ceftazidime	256	256	256–256	16	64	16–256	0.25	64	0.064–256
	Cefoperazone/Sulbactam	32	32	32–32	8	16	4–32	0.125	32	0.016–256
	Ceftriaxone	256	256	256–256	64	128	16–256	0.125	128	0.016–256
	Cefotaxime	256	256	256–256	64	128	16–256	0.125	256	0.016–256
	Cefepime	256	256	256–256	8	8	1–32	0.032	8	0.016–128
	Ertapenem	32	32	32–32	0.032	0.064	0.016–0.125	0.032	0.5	0.016–16
	Imipenem	32	32	32–32	0.25	0.25	0.125–0.25	0.25	1	0.125–2
	Meropenem	32	32	32–32	0.016	0.032	0.016–0.032	0.032	0.064	0.016–4
	Amikacin	256	256	256–256	1	2	1–8	1	2	0.5–256
	Minocycline	4	4	4–4	4	4	2–8	2	4	1–64
	Ciprofloxacin	64	64	64–64	2	32	0.25–64	0.032	4	0.016–64
	Levofloxacin	4	4	4–4	1	8	0.5–16	0.064	4	0.032–16
	Moxifloxacin	8	8	8–8	2	16	1–16	0.125	4	0.032–16

^a^: Of the 15 carbapenem resistant *E.coli*, 5 strains harbored mcr-1 and NDM-5 simultaneously

^b^: Sensitive strains referred to strains do not have ESBL and carbapenem resistance

*K. pneumoniae* strains resistant to tigecycline were susceptible to eravacycline at higher MIC_50_ values of 8 mg/L, while the MIC_90_ was equivalent to that of tigecycline at 16 mg/L. For *mcr-1* positive strains, the MIC_50_ of eravacycline was 1 mg/L compared with 16 mg/L for tigecycline, while the MIC_90_ of eravacycline and tigecycline was equivalent at 16 mg/L. The MIC_50_ (0.5 mg/L) and MIC_90_ (2 mg/L) values of eravacycline against carbapenem-resistant *K. pneumoniae,* were much lower than those of other antibiotics such as imipenem, meropenem, cephalosporins, and fluoroquinolones. The MIC distributions for *K. pneumoniae* of different resistant phenotypes to eravacycline, tigecycline, and other clinically common antibiotics are presented in Table 3.

**Table 3:** MIC distribution of eravacycline and relevant antibiotics against *K. pneumoniae* of different resistance characteristics

Antibiotics	Sensitive, n=10			ESBL, n=10			*kpc*-2 positive, n=9			NDM-1 positive, n=3			*mcr*-1 positive, n=4			Tigecycline resistant, n=13		
	MIC_50_	MIC_90_	Range	MIC_50_	MIC_90_	Range	MIC_50_	MIC_90_	Range	MIC_50_	MIC_90_	Range	MIC_50_	MIC_90_	Range	MIC_50_	MIC_90_	Range
Eravacycline	0.25	0.5	0.125-0.5	0.5	1	0.125-2	0.5	2	0.25-4	0.5	1	0.5-1	1	16	0.5-16	8	16	2-16
Tigecycline	0.5	1	0.5-2	1	4	0.5-4	1	4	0.125	1	2	1--2	16	16	2-16	8	16	8-16
Piperacillin/Tazobactam	2	4	2-4	4	256	2-256	256	256	256-256	256	256	256-256	4	4	4-4	16	32	4-32
Cefoxitin	4	8	2-16	8	16	2-32	256	256	64-256	256	256	256-256	8	8	2-8	32	64	8-128
Ceftazidime	0.125	0.25	0.125-0.25	64	256	16-256	64	256	32-256	256	256	256-256	1	1	0.125-1	1	64	0.5-64
Cefoperazone/Sulbactam	0.25	0.25	0.125-0.25	16	64	8-64	256	256	256-256	256	256	256-256	1	1	0.5-1	2	32	1-128
Ceftriaxone	0.064	0.064	0.032-0.125	256	256	64-256	256	256	16-256	256	256	256-256	0.064	0.125	0.032-0.125	0.25	256	0.064-256
Cefotaxime	0.032	0.125	0.032-0.125	256	256	64-256	256	256	32-256	256	256	256-256	0.125	0.125	0.032-0.125	0.5	128	0.125-256
Cefepime	0.032	0.064	0.032-0.064	32	64	4-128	64	256	32-256	128	256	128-256	2	2	0.032-2	2	64	0.125-64
Ertapenem	0.016	0.016	0.016-0.016	0.25	0.5	0.032-0.5	32	32	32-32	32	32	32-32	0.016	0.016	0.016-0.016	0.032	0.25	0.016-0.5
Imipenem	0.125	0.25	0.125-1	0.125	0.25	0.125-0.25	8	32	8-32	8	32	8-32	0.125	0.25	0.125-0.25	0.125	0.125	0.125-0.5
Meropenem	0.016	0.032	0.016-0.032	0.032	0.064	0.032-0.125	16	32	8-32	16	32	8-32	0.032	0.064	0.032-0.064	0.032	0.064	0.016-0.064
Colistin	0.25	0.25	0.125-0.25	0.25	0.25	0.125-0.25	0.25	0.25	0.125-0.25	0.25	0.25	0.125-0.25	32	64	16-64	0.25	32	0.125-32
Amikacin	1	1	0.5-1	1	4	0.5-32	1	256	0.5-256	2	2	1--2	1	1	1-1	1	2	0.5-256
Minocycline	2	4	2-8	16	32	2-32	32	32	4-32	32	32	4-32	16	32	16-32	32	128	16-256
Ciprofloxacin	0.016	0.032	0.016-0.25	2	64	0.016-64	32	64	16-64	64	64	64-64	32	32	0.032-32	32	64	0.25-64
Levofloxacin	0.064	0.125	0.064-0.5	2	16	0.064-64	16	64	16-64	32	32	16-32	16	16	0.064-16	8	32	0.5-64

^a^: Sensitive strains referred to strains do not have ESBL, carbapenem resistance and polymyxin resistance

MIC distributions for *A. baumannii* also varied by resistance characteristics. *A. baumannii* isolates were tigecycline resistant and showed slightly elevated MIC_50_ and MIC_90_ for eravacycline at 2 mg/L. OXA-23-producing *A. baumannii* isolates have a MIC_50_ of 1 mg/L and MIC_90_ of 2 mg/L for eravacycline, and these values were much lower than the MIC_50_ and MIC_90_ of tigecycline (4 mg/L, 4 mg/L), imipenem (64 mg/L, 64 mg/L), and meropenem (32 mg/L, 64 mg/L). The MIC distributions for *A. baumannii* with different resistant phenotypes to eravacycline, tigecycline, and other clinically relevant antibiotics such as imipenem, meropenem, and colistin are presented in Table 4.

**Table 4:** MIC distribution of Eravacycline and relevant antibiotics against *A. baumannii* of different resistance characteristics

Antibiotics	Sensitive ^a^, n = 9			OXA-23 positive, *n* = 21			Tigecycline resistant, n = 9		
	MIC_50_	MIC_90_	Range	MIC_50_	MIC_90_	Range	MIC_50_	MIC_90_	Range
Eravacycline	0.125	0.25	0.016–0.25	1	2	0.5–2	2	2	2–4
Tigecycline	0.25	0.5	0.25–0.5	4	4	4–8	8	8	8–8
Piperacillin/Tazobactam	2	4	0.016–8	256	256	256–256	256	256	256–256
Ceftazidime	2	8	0.125–32	256	256	64–256	256	256	256–256
Cefepime	1	4	0.032–32	64	256	32–256	256	256	128–256
Imipenem	0.125	1	0.125–1	64	64	16–64	64	64	64–128
Meropenem	0.032	1	0.016–1	32	64	16–64	64	64	32–128
Colistin	0.125	0.25	0.125–0.25	0.25	0.25	0.125–0.25	0.25	0.25	0.25–0.25
Amikacin	4	4	1–4	256	256	256–256	256	256	256–256
Minocycline	0.125	16	0.064–16	8	16	4–16	8	8	8–16
Ciprofloxacin	0.125	0.5	0.032–32	32	32	32–32	32	32	32–32
Levofloxacin	0.125	1	0.064–32	16	32	8–32	16	16	16–32

^a^: Sensitive strains referred to strains do not have carbapenem resistance and tigecycline resistance

For *S. maltophilia* there is no breakpoints available for tigecycline, the MIC distributions of tigecycline and eravacycline against *S. maltophilia* were evaluated. The MIC_50_ and MIC_90_ for eravacycline were both 1 mg/L, at the same time the MIC_50_ and MIC_90_ for tigecycline were 0.5 mg/L and 1 mg/L.

For *Staphylococcus* spp*.*, the results indicated that MIC_50_ and MIC_90_ of eravacycline were 0.25 mg/L and 0.5 mg/L, respectively, for MRSA (methicillin-resistant *S. aureus*), for MSSA (methicillin-sensitive *S. aureus*) the MIC_50_ of eravacycline was as low as 0.064 mg/L, and MIC_90_ remained the same as that of MRSA. MIC_50_ and MIC_90_ of eravacycline for methicillin-resistant coagulase-negative *staphylococci* (MRCoNS) were 0.25 mg/L and 1 mg/L, respectively, and for MSCoNS (methicillin-sensitive coagulase-negative *staphylococci*) the values of eravacycline were lower at 0.016 mg/L and 0.25 mg/L, respectively. For other antibiotics, the values are presented in Table 5.

**Table 5:** MIC distribution of Eravacycline and relevant antibiotics against *Staphylococcus. spp* of different resistance characteristics

Antibiotics		MRSA^a^, *N* = 15		MSSA^b^, *N* = 6			MRCoNS^c^, *N* = 24				MSCoNS^d^, N = 15		
	MIC_50_	MIC_90_	Range	MIC_50_	MIC_90_	Range	MIC_50_	MIC_90_		Range	MIC_50_	MIC_90_	Range
Eravacycline		0.25	0.5	0.032–1	0.064	0.5	0.016–2	0.25	1	0.016–2	0.016	0.25	0.008–0.25
Tigecycline		0.25	0.5	0.125–0.5	0.25	0.25	0.125–0.25	0.25	0.5	0.125–0.5	0.125	0.25	0.064–0.25
Oxacillin		64	64	2–64	0.25	0.5	0.25–0.5	2	64	0.5–256	0.125	0.25	0.125–0.25
Cefoxitin		256	256	32–256	4	4	2–4	16	256	2–256	2	8	1–8
Vancomycin		1	1	0.5–1	0.5	0.5	0.5–0.5	1	2	0.5–2	0.5	1	0.25–1
Teicoplanin		2	2	0.5–2	0.5	0.5	0.5–1	2	4	0.064–8	0.5	2	0.125–2
Erythromycin		256	256	0.25–256	256	256	0.25–256	64	256	0.125–256	0.25	256	0.064–256
Minocycline		4	16	0.064–32	0.064	0.125	0.064–0.125	0.25	0.5	0.064–8	0.125	0.25	0.064–0.5
Ciprofloxacin		64	64	0.25–64	0.5	0.5	0.25–0.5	16	64	0.125–64	0.25	8	0.125–64
Levofloxacin		32	64	0.25–64	0.25	0.25	0.125–0.5	4	128	0.25–128	0.25	0.5	0.125–128
Moxifloxacin		8	16	0.016–32	0.032	0.064	0.016–0.064	1	16	0.064–32	0.064	1	0.032–16
Trimethoprim/Sulfamethoxazole		0.125	16	0.032–16	0.032	0.064	0.032–0.25	4	32	0.064–64	0.125	4	0.016–4
Chloramphenicol		8	8	4–32	8	8	4–64	4	8	2–64	4	4	2–8
Rifampin		256	256	0.004–256	0.008	0.016	0.004–0.016	0.008	256	0.004–256	0.008	0.016	0.004–0.016
Clindamycin		128	256	0.064–256	0.064	256	0.064–256	0.125	256	0.064–256	0.064	0.125	0.064–0.25
Linezolid		1	2	0.5–2	1	2	1–2	1	1	0.5–1	1	1	0.5–2

^a^ Methicillin-resistant *Staphylococcus aureus*. ^b^ Methicillin- sensitive *Staphylococcus aureus*

^c^ Methicillin-resistant coagulase-negative *staphylococci*. ^d^ Methicillin- sensitive coagulase-negative *staphylococci*

In the results obtained for *Enterococcus* spp*.* it was found that MIC_50_ and MIC_90_ of eravacycline for *E. faecalis* were both 0.032 mg/L. The MIC_50_ and MIC_90_ of eravacycline for *E. faecium* were 0.016 mg/L and 0.032 mg/L. For Vancomycin-Resistant *Enterococci* (VRE) strains, the MIC_50_ and MIC_90_ were identical with that of vancomycin-susceptible *E. faecium* strains. For other antibiotics, the values are presented in Table 6. In general, for gram-positive bacteria with varying resistance factors, eravacycline demonstrated substantial antibacterial activity.

**Table 6:** MIC distribution of Eravacycline and relevant antibiotics against *Enterococci. spp* of different resistance characteristics

Antibiotics	*E.faecalis*, *n* = 10			*E.faecium*, *n* = 8			VRE^a^, n = 3		
	MIC_50_	MIC_90_	Range	MIC_50_	MIC_90_	Range	MIC_50_	MIC_90_	Range
Eravacycline	0.032	0.032	0.016–0.125	0.016	0.032	0.008–0.064	0.016	0.032	0.008–0.032
Tigecycline	0.064	0.064	0.064–0.125	0.064	0.064	0.016–0.125	0.125	0.25	0.125–0.25
Ampicillin	1	8	1–8	64	64	4–64	64	64	64–64
Vancomycin	1	2	0.5–2	0.5	1	0.25–1	128	128	128–128
Teicoplanin	0.125	0.25	0.032–0.25	0.25	0.25	0.064–0.25	32	64	32–64
Erythromycin	1	256	0.25–256	256	256	0.016–256	0.125	256	0.125–256
Minocycline	16	16	0.064–16	0.032	16	0.032–16	0.064	16	0.064–16
Ciprofloxacin	2	32	0.5–64	64	64	4–64	64	64	64–64
Levofloxacin	2	64	1–64	64	128	1–128	64	64	64–64
Linezolid	1	2	1–2	1	1	0.5–1	1	1	1–1

^a^ VRE referred to vancomycin-resistant *Enterococci*. All of the 3 VRE strains in this study were *E.faecium*

For fastidious strains, including 20 *S. pneumoniae* isolates and 20 *H. influenzae* isolates, eravacycline showed high antimicrobial activities against *S. pneumoniae* with MIC_50_ (0.008 mg/L) and MIC_90_ (0.008 mg/L), there was no difference with eravacycline distribution between PRSP (Penicillin-resistant *S. pneumoniae*) and PSSP (Penicillin-sensitive *S. pneumoniae*) strains (Table 7). For *H. influenzae* the MIC_50_ and MIC_90_ were 0.064 mg/L and 0.125 mg/L, and they were the same in both β-lactamase-positive and β-lactamase-negative strains (Table 8).

**Table 7:** MIC distribution of Eravacycline and relevant antibiotics against *S.pneumoniae* of different resistance characteristics

Antibiotics	PSSP^a^, n = 10			PRSP^b^, n = 10		
	MIC_50_	MIC_90_	Range	MIC_50_	MIC_90_	Range
Eravacycline	0.008	0.008	0.002–0.016	0.008	0.008	0.004–0.008
Tigecycline	0.016	0.016	0.008–0.016	0.016	0.016	0.016–0.016
Penicillin	0.016	0.016	0.016–0.032	4	4	4–4
Amoxicillin/Clavulanic acid	0.016	0.064	0.008–0.25	8	8	8–8
Cefuroxime	0.032	0.125	0.016–0.5	16	32	8–32
Cefaclor	1	2	1–4	256	256	128–256
Ceftriaxone	0.032	0.064	0.016–0.125	2	8	1–8
Erythromycin	8	32	0.5–256	256	256	128–256
Azithromycin	16	32	4–256	256	256	256–256
Clindamycin	0.125	128	0.032–256	256	256	128–256
Clarithromycin	2	32	0.25–256	256	256	256–256
Levofloxacin	1	1	0.25–32	1	1	1–1
Moxifloxacin	0.125	0.125	0.064–16	0.125	0.25	0.125–0.25
Trimethoprim/Sulfamethoxazole	4	8	0.064–8	8	16	4–32
Tetracycline	32	64	4–64	32	32	32–32
Chloramphenicol	4	8	1–16	4	4	4–4
Vancomycin	0.25	0.25	0.125–0.25	0.25	0.25	0.25–0.25

^a^ *PSSP* Penicillin-sensitive *Streptococcus pneumoniae*

^b^ *PRSP* Penicillin-resistant *Streptococcus pneumoniae*

**Table 8:** MIC distribution of Eravacycline and relevant antibiotics against *H. influenza* of different resistance characteristics

Antibiotics	β-lactamases negative, n = 10			β-lactamases positive, n = 10		
	MIC_50_	MIC_90_	Range	MIC_50_	MIC_90_	Range
Eravacycline	0.064	0.125	0.064–0.125	0.064	0.125	0.032–0.125
Tigecycline	0.25	0.5	0.125–0.5	0.125	0.25	0.064–0.5
Ampicillin	0.125	0.5	0.125–1	16	64	0.064–64
Amoxicillin/Clavulanic acid	0.125	0.5	0.125–0.5	1	1	0.5–1
Penicillin	16	32	0.032–32	16	32	1–64
Cefaclor	2	8	0.5–8	4	16	1–32
Cefuroxime	1	2	0.25–4	1	4	0.25–16
Azithromycin	1	4	0.064–4	2	64	0.25–64
Clarithromycin	4	16	0.5–16	4	64	1–64
Levofloxacin	0.032	1	0.016–1	0.032	0.125	0.016–0.5
Moxifloxacin	0.032	1	0.016–1	0.032	0.25	0.016–0.5
Trimethoprim/Sulfamethoxazole	16	32	0.032–32	16	32	1–64
Tetracycline	1	4	0.064–4	2	64	0.25–64
Chloramphenicol	0.5	1	0.25–1	1	8	0.5–8

**Fig. 1:**
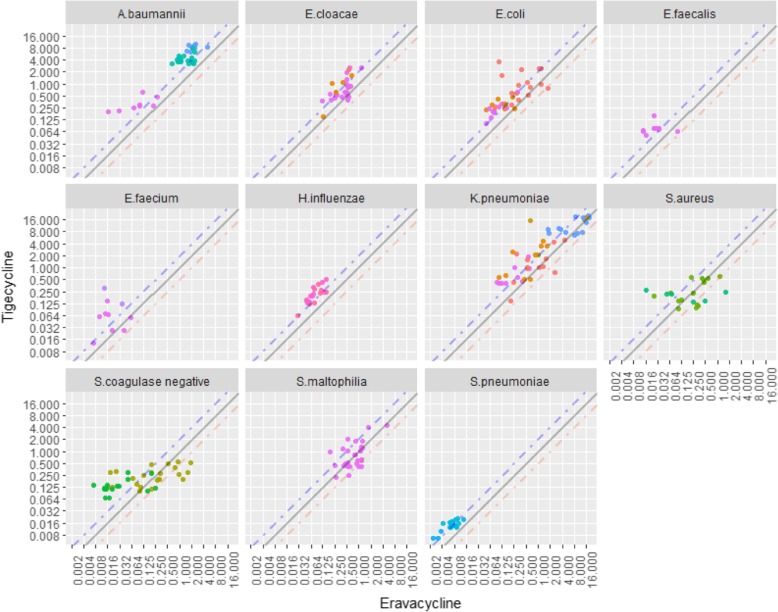
Scatter plot of MIC values of tigecycline versus MIC value of eravacycline against various bacteria. Note: A tiny displacement was made to the points in this figure in order to avoid overlapping of the strains with the same eravacycline and tigecycline MIC values. This tiny displacement can ensure the actual distribution of all strains visible. The points on the grey solid line indicated the strains shared the identical eravacycline and tigecycline MIC values. The points above the blue dash line indicated that the MIC values of tigecycline were greater than twice than the MIC values of eravacycline. The points below the orange dash line indicated that the MIC values of eravacycline were greater than twice than the MIC values of tigecycline Legends:  Carbapenem resistant;  ESBL;  mcr-1;  MRCoNS;  MRSA;  MSCoNS;  MSSA;  OXA-23;  PRSP;  PSSP;  Tigecycline resistant;  VRE;  without resistance gene;  β-lactamases –;  β-lactamases +.

A jittered scatter plot was drawn using the MIC values of eravacycline and tigecycline involving all the strains tested. A clear pattern was found showing that most of the MIC values of tigecycline are higher than the corresponding MIC values of eravacycline (in many cases by 2 to 4 fold). For all of the clinical isolates tested, except for *Staphylococcus* spp. and *S. maltophilia,* more points are located above the diagonal y = x line, suggesting that eravacycline has lower MIC distribution than tigecycline (Fig. 1). For *Staphylococcus* spp. and *S. maltophilia* the points were distributed on both sides of the diagonal evenly, suggesting a comparable MIC distribution between eravacycline and tigecycline.

## Discussion

As resistance to antibiotics grows worldwide, it becomes increasingly important to find new treatments for bacterial infections. In the present study, a new antibiotic eravacycline was compared to existing medications. Eravacycline demonstrated high in vitro activity against clinical isolates, including strains with specific resistant factors. Eravacycline was compared to a derivative of tigecycline, and in most cases presented with a lower MIC distribution for the majority of strains tested in this study. Since many years nosocomial pathogens, such as *Enterobacteriaceae* which are responsible for complicated intra-abdominal infection (cIAI) were increasing in frequency [15]. Moreover, cases of gram-positive cocci such as *S. aureus*, coagulase-negative *staphylococci,* and *enterococci,* the major causative organisms of complicated urinary tract infections (cUTI) were also increasing [16]. The emergence of multiple drug-resistant bacteria, such as Carbapenem resistant *Enterobacteriaceae* bacteria (CRE), Carbapenem-resistant *Acinetobacter baumannii* (CRAB) and Methicillin-resistant *Staphylococcus aureus* (MRSA), has compounded this problem significantly by increasing the difficulty of treatment, the proportion of failures, as well as the mortality rate of patients. Since Tigecycline and eravacycline belong to a different antibiotic class with a mechanism of action distinct from cephalosporins and carbapenem antibiotics, they can evade established resistance mechanisms of *Enterobacteriaceae* and exhibit higher efficacy against resistant bacteria. In this study, eravacycline showed high antibacterial activity against CRE strains, suggesting that eravacycline could be useful to treat complicated infections caused by CRE. Similarly, CRAB also shows resistance to antibiotics which were commonly used during the clinical practice. CRAB is the most notorious pathogen responsible for nosocomial infections in China at present [17–19]. This study found that the most effective drug for OXA-23 producing *A. baumannii* was colistin then eravacycline. Eravacycline also demonstrated high potency against OXA-23 producing *A. baumannii*, with a MIC_50_ of 1 mg/L which was much lower than other antibiotics, except for colistin. Similar to eravacycline in structure and mechanism, tigecycline has been widely utilized in China for many years, and tigecycline-resistant strains have also emerged with the increase in use of this antibiotic [20, 21]. In the present study, eravacycline also exhibited lower MIC distribution compared with tigecycline in tigecycline-resistant strains, suggesting that the mechanism which leads to tigecycline resistance does not inhibit the activity of eravacycline. Furthermore, high antibiotic potency against CRE and CRAB could make eravacycline a potential option to treat complex infections including respiratory and bloodstream infections. For *Staphylococcus* spp*.* the results were entirely different, with tigecycline values much lower than eravacycline. From the scatter plot we observed that the points are evenly distributed on both sides of the diagonal line (line: *y = x*). This may be either due to the combined effects of different resistance mechanisms, or potentially unknown resistance mechanisms. In addition, the total number of *Staphylococcus* spp. strains which were tested in this study was relatively small, which may cause random errors in the antibacterial activity of eravacycline. Thus, further validation utilizing different bacterial isolates is required. For fastidious strains, eravacycline demonstrated excellent potency despite resistance characteristics of the strains. From the scatter plot, we can see that although MIC values of eravacycline were generally lower than those of tigecycline, the MIC values of eravacycline were also rising with the MIC values of tigecycline proportionally, thus, we need to be alert to the possible cross-resistance potential of eravacycline and tigecycline, especially in strains with higher MIC values of tigecycline.

### Limitation and suggestion

The clinical isolates tested were limited by country as they were exclusively collected in China and within this country, these isolates were only obtained from 11 teaching hospitals. No strains from other hospitals were utilized. Therefore, many different clinical isolates remain untested. Thus, it is important that researchers reproduce our work in other countries with different isolates in order to understand the full spectrum of this new antibiotics’ efficacy. The results of this study show that eravacycline has a positive application potential for the treatment of current drug-resistant bacterial infections. Considering the relatively small number of each organism and limited types of resistant phenotypes, the result of this study only partially represent the resistant phenotype encountered in real clinical practice, and additional studies are needed for a more comprehensive assessment of the antibacterial activity of eravacycline.

## Conclusions

The results of this study proved that eravacycline possesses a broad spectrum of activity against a variety of gram-positive and gram-negative bacteria, including multi-drug resistant strains such as *A. baumannii* and carbapenem-resistant *Enterobacteriaceae.*

## Additional file

Additional file 1: The list of committee and the institute to which it belongs for all hospitals that provided Administrative Consent to access or receive samples. This additional file list the committee (and the institute to which it belongs) for all hospitals that provided Administrative Consent to access or receive samples/data (DOCX 13 kb)

## Data Availability

The datasets used and analyzed during the current study are available from the corresponding author upon reasonable request.

## Abbreviations

CLSI: Clinical and Laboratory Standards Institute
CRAB: Carbapenem resistant *Acinetobacter baumannii*
CRE: Carbapenem resistant *Enterobacteriaceae*
cUTI: complicated urinary tract infections
ESBL: extended-spectrum-lactamases
MIC: minimum inhibitory concentration
MRSA: methicillin-resistant *Staphylococcus aureus*
MSCoNS: Methicillin- sensitive coagulase-negative *staphylococci*
PCR: polymerase chain reaction
PRSP: penicillin resistant *Streptococcus pneumoniae*
VRE: Vancomycin-resistant *enterococci*
