# Supplementary material for: In vitro activities of Eravacycline against 336 isolates collected from 2012 to 2016 from 11 teaching hospitals in China
Source: BMC Infect Dis. 2019 Jun 10;19:508. doi: 10.1186/s12879-019-4093-1 (PMC6558774; doi:10.1186/s12879-019-4093-1)
Supplement: Supplementary file 1 — The list of committee and the institute to which it belongs for all hospitals that provided Administrative Consent to access or receive samples. This additional file list the committee (and the institute to which it belongs) for all hospitals that provided Administrative Consent to access or receive samples/data (DOCX 13 kb) [file 12879_2019_4093_MOESM1_ESM.docx]

The list of committee and the institute to which it belongs for all hospitals that provided Administrative Consent to access or receive samples

| NO. | Name of hospitals | Department | Behavior |
| --- | --- | --- | --- |
| 1 | Peking University People's Hospital | Ethics committee | Research project approval |
|  |  | Scientific research department | grant permissions to access the raw samples |
| 2 | Zhongshan Hospital affiliated to Fudan University | Scientific research department | grant permissions to access the raw samples |
| 3 | Guangzhou Respiratory Disease Research Institute | Scientific research department | grant permissions to access the raw samples |
| 4 | The First Affiliated Hospital of China Medical University | Scientific research department | grant permissions to access the raw samples |
| 5 | Tianjin General Hospital | Scientific research department | grant permissions to access the raw samples |
| 6 | Xijing Hospital, Fourth Military Medical University | Scientific research department | grant permissions to access the raw samples |
| 7 | Second Affiliated Hospital of Zhejiang University School of Medicine | Scientific research department | grant permissions to access the raw samples |
| 8 | The First Affiliated Hospital of Xiangya School of Medicine | Scientific research department | grant permissions to access the raw samples |
| 9 | Chaoyang Hospital, Capital Medical University | Scientific research department | grant permissions to access the raw samples |
| 10 | Wuhan Puai Hospital | Scientific research department | grant permissions to access the raw samples |
| 11 | Shandong Provincial Hospital | Scientific research department | grant permissions to access the raw samples |
